# Supplementary material for: Fecal Microbiota and Hair Glucocorticoid Concentration Show Associations with Growth during Early Life in a Pig Model
Source: Nutrients. 2022 Nov 3;14(21):4639. doi: 10.3390/nu14214639 (PMC9655727; doi:10.3390/nu14214639)
Supplement: Supplementary file 1 [file nutrients-14-04639-s001.zip › nutrients-1983624-Supplementary.pdf]

Supplementary Table S1. Mean body weight (BW) and average daily gain (ADG) of the animals included in each group for lactation and nursery periods. Each mean is followed by its

Group	Fast Fast	Fast Slow	Slow Fast	Slow Slow	DF	F value	P-value
Lactation ADG, g/d	246 ± 5.2 a	244 ± 4.6 a	148 ± 3.3 b	138 ± 5.9 b	71	151.4	< 0.001
Nursery ADG, g/d	273 ± 7.1 a	128 ± 3.7 c	238 ± 5.7 b	112 ± 3.5 d	71	203.5	< 0.001
Birth BW, kg	1.45 ± 0.026 a	1.31 ± 0.069 ab	1.35 ± 0.074 ab	1.21 ± 0.056 b	71	2.904	0.040
Lactation BW, kg	6.7 ± 0.083 a	6.4 ± 0.094 a	4.5 ± 0.105 b	4.1 ± 0.112 b	71	123.6	< 0.001
Nursery BW, kg	17.9 ± 0.330 a	11.7 ± 0.196 c	14.2 ± 0.306 b	8.7 ± 0.170 d	71	215.1	< 0.001

Fast_Fast: Pigs showing fast growth during lactation and nursery periods.

Fast_Slow: Pigs showing fast growth during lactation period and slow growth during nursery period.

Slow_Fast: Pigs showing slow growth during lactation period and fast growth during nursery period.

Slow_Slow: Pigs showing slow growth during lactation and nursery periods.

DF: Degrees of freedom.

a,b,c,d Values with different letter in the same column are significantly different according to ANOVA and Tukey adjust.

Supplementary Table S2. Hair glucocorticoid concentration, alpha diversity and SCFA concentration for each sex, lactation growth group and nursery growth group.

.	Lactation growth			Nursery growth			Sex				*F* value				*P* value			
Item	Fast	Slow	SEM	Fast	Slow	SEM	Females	Males	SEM	DF	Lactation growth	Nursery growth	Lactation x nursery growth	Sex	Lactation growth	Nursery growth	Lactation x nursery growth	Sex
Cortisol, pg/mg	18.3	19.7	0.74	19.7	152.9	0.388	19.1	18.8	0.77	68	2.29	0.54	0.03	0.52	0.130	0.463	0.852	0.472
Cortisone, pg/mg	140	109.6	22.3	18.200	98.900	0.6020	135	118	22.8	53	0.95	4.18	1.85	0.47	0.329	0.041	0.174	0.492
Cortisol/cortisone	0.40	0.62	0.115	0.74	23.40	0.110	0.48	0.51	0.112	53	1.46	3.30	2.00	0.40	0.227	0.069	0.157	0.527
Observed OTUs	354	343	14.9	353	345	15.1	346	352	15.2	64	0.95	0.00	0.40	1.26	0.330	0.969	0.526	0.260
Eveness	0.786	0.781	0.0078	0.797	0.770	0.0074	0.778	0.790	0.0075	64	0.33	5.79	0.00	0.97	0.604	0.018	0.959	0.325
Shannon	6.63	6.55	0.109	6.72	6.46	0.105	6.55	6.64	0.107	64	0.76	2.32	0.01	0.69	0.411	0.137	0.929	0.407
Total SCFA, µmol/g	84.02	75.54	2.970	79.68	80.35	2.930	80.2	79.8	2.98	63	4.63	0.03	1.03	0.00	0.031	0.864	0.310	0.986
Acetate, µmol/g	47.33	42.39	1.848	43.56	46.17	1.823	45.5	44.5	1.86	63	3.85	0.95	1.43	0.07	0.049	0.331	0.231	0.797
Butyrate, µmol/g	10.20	8.05	0.589	9.67	8.58	0.581	9.0	9.3	0.60	63	7.53	5.86	0.00	0.14	0.006	0.017	0.964	0.711

DF: Degrees of freedom of the statistical test.
